# Association Between Serum Cystatin C and Thyroid Diseases: A Systematic Review and Meta-Analysis

**DOI:** 10.3389/fendo.2021.766516

**Published:** 2021-11-19

**Authors:** Caihong Xin, Jing Xie, Huaying Fan, Xin Sun, Bimin Shi

**Affiliations:** ^1^ Department of Endocrinology and Metabolism, Fourth People’s Hospital of Shenyang, Shenyang, China; ^2^ Department of Endocrinology and Metabolism, First Affiliated Hospital of Soochow University, Suzhou, China

**Keywords:** cystatin C, CysC, thyroid disease, systematic review, meta-analysis

## Abstract

**Background:**

Cystatin C (CysC) is often used to diagnose and monitor renal diseases. Although some studies have investigated the association between serum CysC levels and thyroid diseases, their reported results were inconsistent. Therefore, the relationship between CysC levels and thyroid diseases remains controversial.

**Aim:**

This meta-analysis aimed to statistically evaluate serum CysC levels in patients with thyroid diseases.

**Methods:**

A literature search was conducted using the PubMed, Web of Science, Embase, EBSCO, and Wiley Online Library databases. The following search terms were used for the title or abstract: “Cystatin C” or “CysC” in combination with the terms “thyroid disease”, “thyroid function”, “hypothyroidism”, or “hyperthyroidism”. The results of the systematic analysis were presented as standardized mean differences (SMDs) with corresponding 95% confidence intervals (CIs).

**Results:**

Eleven articles (1,265 cases and 894 controls) were included in the meta-analysis. The results of the meta-analysis showed that the serum CysC levels of patients with hyperthyroidism were significantly higher than those of the controls (SMD: 1.79, 95% CI [1.34, 2.25]), and the serum CysC levels of patients with hypothyroidism were significantly lower than those of the controls (SMD −0.59, 95% CI [−0.82, −0.36]). Moreover, the treatment of thyroid diseases significantly affected serum CysC levels.

**Conclusions:**

To the best of our knowledge, this meta-analysis is the first to evaluate serum CysC levels in patients with thyroid diseases. Our findings suggest that thyroid function affects serum CysC levels and that serum CysC may be an effective marker for monitoring thyroid diseases.

**Systematic Review Registration:**

PROSPERO [https://www.crd.york.ac.uk/PROSPERO/display_record.php?RecordID=258022], identifier CRD42021258022].

## Introduction

Cystatin C (CysC), a small-molecule protein belonging to the cysteine protease inhibitor superfamily, is produced by all nucleated cells. Compared with serum creatinine, changes in CysC levels can sensitively reflect changes in the glomerular filtration rate (GFR). Therefore, it is often used in the diagnosis and evaluation of kidney diseases (1). Studies have shown that serum CysC is a sensitive biomarker for detecting changes in GFR and identifying mild kidney diseases (2, 3). Moreover, patients with diabetes, chronic obstructive pulmonary disease, ischemic stroke, and myocardial infarction have been reported to have higher serum CysC levels than healthy individuals (4–7).

Thyroid diseases, including hyperthyroidism and hypothyroidism, are common endocrine system diseases. Thyroid hormones have a great influence on renal hemodynamics, water–salt balance, ion transport, renal tubular secretion, and reabsorption (8). Few studies have investigated the association between serum CysC levels and thyroid diseases. Some authors reported that the serum CysC levels of patients with hyperthyroidism and hypothyroidism were higher and lower, respectively, although the results were inconsistent (9–11). Therefore, the relationship between serum CysC levels and thyroid diseases remains controversial. This meta-analysis aimed to statistically evaluate serum CysC levels in patients with thyroid diseases. In addition, we used serum CysC levels for treatment monitoring.

## Methods

### Search

We searched the following electronic databases: Web of Science, Embase, PubMed, EBSCO, and Wiley Online Library databases. The following search terms were used for the title or abstract: “Cystatin C” or “CysC” in combination with the terms “thyroid disease”, “thyroid function”, “hypothyroidism”, or “hyperthyroidism”. All studies published between 1980 and 2021 were included in the search. In addition, the references of the retrieved articles were examined to identify additional eligible studies, excluding unpublished studies. The completed Preferred Reporting Items for Systematic Reviews and Meta-Analyses checklist is presented in Supplementary Data (**Table S1**). This systematic review and meta-analysis was registered in PROSPERO (registration number: CRD42021258022).

### Inclusion Criteria

The studies included in this meta-analysis met the following criteria: (1) a case–control or cohort design; (2) detailed data about serum CysC levels in patients with thyroid disease and controls; and (3) published in English.

### Data Extraction and Risk of Bias

Two researchers independently extracted general information from the included articles, such as the first author, publication year, study period, region, study design, and details of cases and controls. The Newcastle–Ottawa Scale is a risk assessment tool for observational studies recommended by the Cochrane Collaboration (12, 13). Quality of evidence was also assessed using the Grade of Recommendations Assessment, Development, and Evaluation (GRADE) approach (14). The two researchers independently assessed the studies through discussion, compared their findings, and resolved any differences by consensus. If no consensus was reached, a third researcher resolved the difference.

### Statistical Analysis

The results of the systematic analysis were presented as standardized mean differences (SMDs) with corresponding 95% confidence intervals (CIs). Heterogeneity among studies was assessed using Cochran’s Q test and I^2^ statistic. I^2^ of <50% was considered to have low or moderate heterogeneity, and a fixed-effects model was used. Otherwise, heterogeneity was considered high, and a random-effects model was used for the analysis. We additionally performed a sensitivity analysis to evaluate the influence of any given study on the pooled estimate. Publication bias was evaluated using Egger’s test. A *P*-value of <0.05 was considered to indicate statistical significance. All statistical analyses were performed using Stata version 12.0 (College Station, TX, USA).

## Results

**Figure 1:**
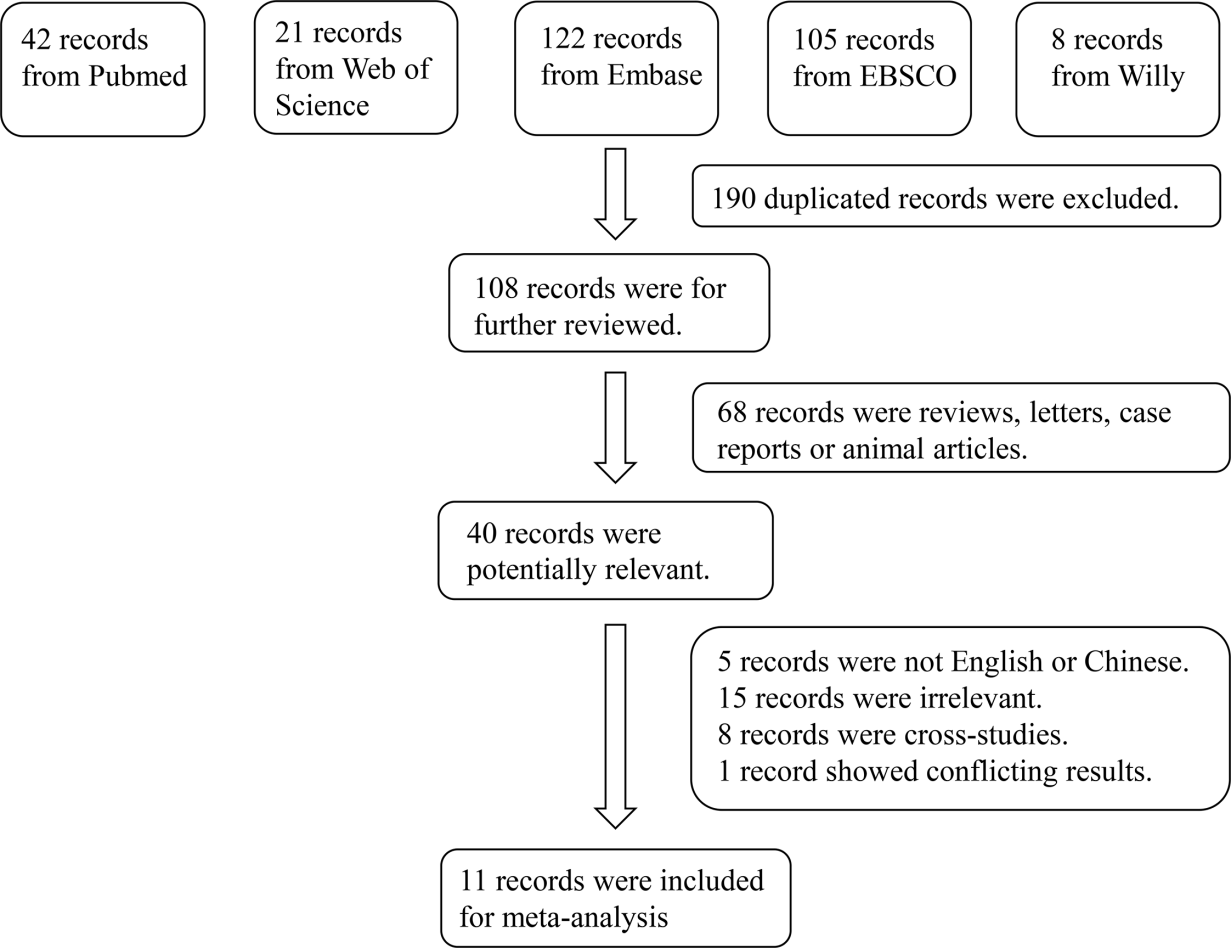
Flowchart of the detailed procedure for the inclusion or exclusion of selected studies.

In total, 298 studies were retrieved from the PubMed, Web of Science, Embase, EBSCO, and Wiley Online Library databases. No articles from the reference lists were included in this study. After screening, 11 articles comprising 1,265 cases and 894 controls were selected (9, 15–24). The inclusion criteria for full-text selection are presented in **Figure 1**. The characteristics of the selected studies are summarized in **Table 1**.

**Table 1:** Study characteristics of the published studies included in the meta-analysis.

Author	Publication Year	Study Period	Region	Study design	Cystatin C level		Case factor
					Case	Control	
Fricker M	2003	2000 – 2002	Switzerland	Prospective study	hyperthyroidism (before treatment): 1.32 ± 0.17; hypothyroidism (before treatment): 0.84 ± 0.17	hyperthyroidism (after treatment): 0.95 ± 0.19; hypothyroidism (after treatment): 1.1 ± 0.28	Patients with newly diagnosed hypo- or hyperthyroidism who had been referred to the Hospital. Hypothyroidism: nine cases. Median age (range) at diagnosis was 34 (14-52) years. hyperthyroidism: thirteen cases. The median age (range) at diagnosis was 43 (22 to 86) years.
Wiesli P	2003	2000 – 2003	Switzerland	Prospective study	hyperthyroidism (before treatment): 1.04 ± 0.29; hypothyroidism (before treatment): 0.88 ± 0.23	hyperthyroidism (after treatment): 0.92 ± 0.25; hypothyroidism (after treatment): 1.01 ± 0.21	Patients with mild thyroid dysfunction who had been referred to the Hospital. Twenty-six patients with subclinical hypothyroidism were included. Median (range) age at diagnosis was 42 (14–78) years. Fourteen patients with subclinical hyperthyroidism were included. The median age (range) at diagnosis was 43 (22–78) years.
Manetti L	2005	2003	Italy	Case-control study	hyperthyroidism: 0.9 ± 0.24; hypothyroidism: 0.69 ± 0.17	0.81 ± 0.17	58 patients with untreated Graves’ disease (47 females and 11 males; 41 ± 13 years) and 20 patients with subclinical hypothyroidism (16 females and 4 males; 37 ± 13 years) were enrolled. The control group had 5 healthy subjects (3 females and 2 males) with age of 41 ± 15 years.
Goede DL	2009	1998 – 2016	Switzerland	Prospective study	0.79 ± 0.27	1.03 ± 0.42	Sixteen Patients with newly diagnosed primary and central hypothyroidism were included in the study. Mean age at diagnosis was 44 ± 18 years. All patients were treated with levothyroxine for 4 ± 2 months.
Özden TA	2010	July 2012 – April 2013	Turkey	Case-control study	0.6 ± 0.1	0.67 ± 0.1	A total of 25 patients with permanent congenital hypothyroidism were included in the study group. Twenty-one age-matched healthy children formed the control group.
Kotajima N	2010	2003 – 2005	Japan	Case-control and prospective study	hyperthyroidism: 0.950 ± 0.188; hypothyroidism: 0.655 ± 0.243	control: 0.732 ± 0.008; hyperthyroidism (after treatment): 0.77 ± 0.17	Thirty-three patients with untreated Graves’ disease (24 females and nine males; 43.7 ± 16.5 years) and eight patients with untreated hypothyroidism (seven females and one male; 52.2 ± 20.6 years) were enrolled. The control group had 25 healthy subjects (17 females and eight males) with age of 41.2 ± 10.2 years.
Stojanoski S	2011	January 2007 – December 2009	Turkey	Case-control and Prospective study	hyperthyroidism (before treatment): 1.65 ± 0.5; hypothyroidism (before treatment): 0.88 ± 0.7	control: 0.85 ± 0.14; hyperthyroidism (after treatment): 0.96 ± 0.5; hypothyroidism (after treatment): 1.24 ± 0.5	Thirty-five consecutive patients (26 females and 9 males; 43 ± 11 years) were enrolled in the study. The study group included: 20 patients (14 females and 6 males) with newly diagnosed hypothyroidism and 15 patients (12 females and 3 males) with newly diagnosed hyperthyroidism. Thirty-five age- and sex-matched normal subjects served as controls.
Kimmel M	2012	–	Germany	Prospective study	hyperthyroidism (before treatment): 1.07 ± 0.21; hypothyroidism (before treatment): 0.8 ± 0.09	hyperthyroidism (after treatment): 0.82 ± 0.08; hypothyroidism (after treatment): 0.88 ± 0.09	Hypothyroidism: nine cases (3 females and 6 males; 42 ± 14 years). hyperthyroidism: seven cases (6 females and 1 male; 46 ± 11 years).
Suzuki Y	2015	March 2013 – September 2014	Japan	Case-control and Prospective study	hyperthyroidism: 1.06 ± 0.20; hyperthyroidism (before treatment): 0.99 ± 0.17	control: 0.82 ± 0.08; hyperthyroidism (after treatment): 0.75 ± 0.06	113 patients with untreated or poorly controlled Graves’ disease (89 females and 24 males; 44.9 ± 14.8 years). The control group had 146 age-matched healthy volunteers subjects (100 females and 46 males) with age of 49.2 ± 18.1 years.
Al Musaimi O	2019	–	Saudi Arabia	Case-control study	hyperthyroidism: 4.14 ± 2.69; hypothyroidism: 0.60 ± 0.46	1.15 ± 0.40	9 patients with thyroid hypothyroidism (four cases) and hyperthyroidism (five cases) dysfunctions (6 females and 1 male) with an age interval of (28–61) years. The control group had 16 healthy subjects (3 females and 13 males) with median age (range) was 43 (22–78) years.
Can N	2020	–	Turkey	Case-control study	1.35 ± 0.22	0.74 ± 0.09	Thirty patients with Graves’ disease (13 females and 17 males; 36.1 ± 18.4 years). The control group had 30 healthy subjects (14 females and 16 males) with age of 34.0 ± 10.1 years.

### Results of the Meta-Analysis

**Figure 2:**
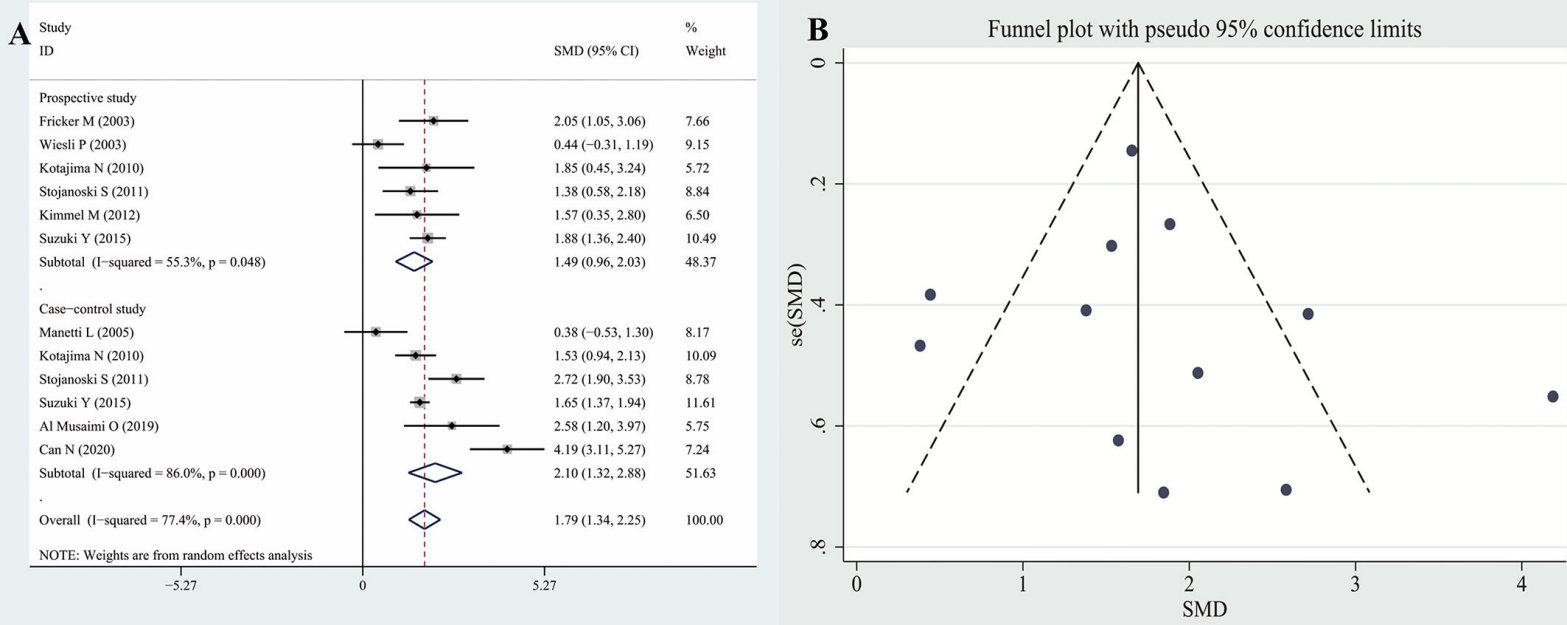
Forest plots and funnel plots of serum CysC in patients with hyperthyroidism compared with controls. Diamond represents the SMDs at 95% CI. **(A)** Forest plots; **(B)** Funnel plots. CysC, Cystatin C; SMD, standardized mean difference; CI, confidence interval.

**Figure 3:**
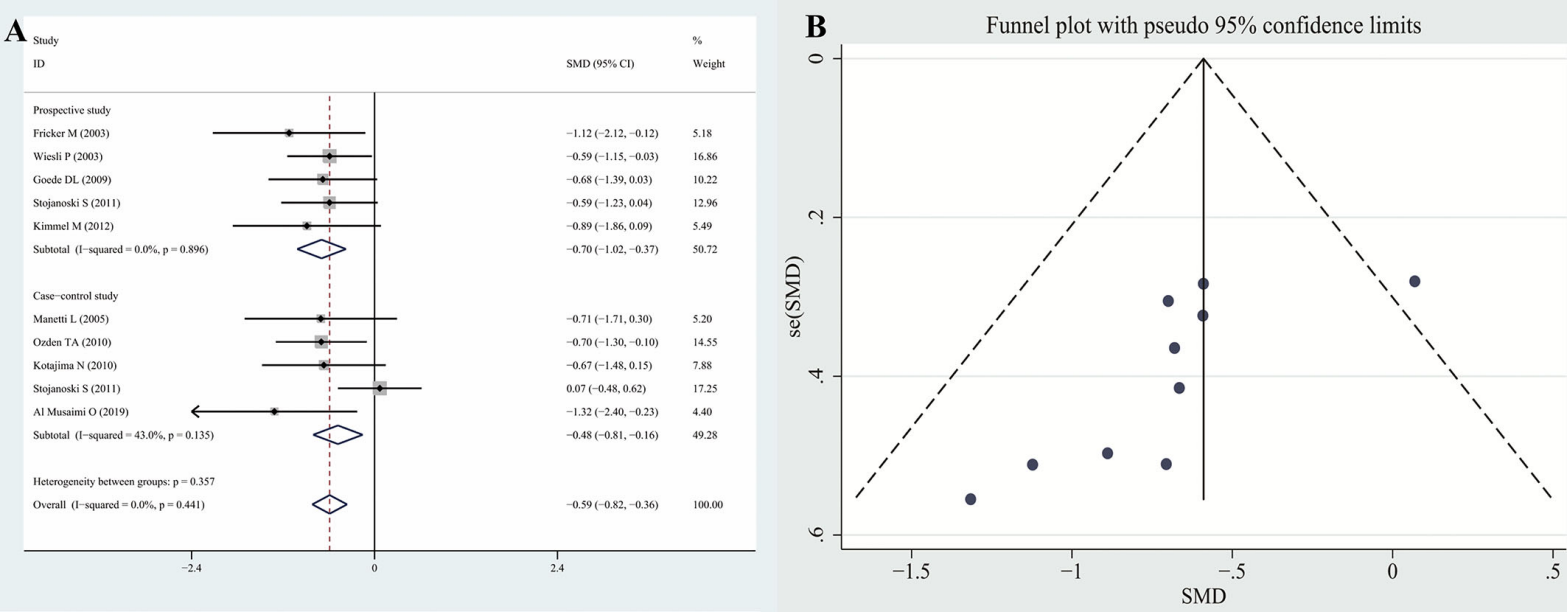
Forest plots and funnel plots of serum CysC in patients with hypothyroidism compared with controls. Diamond represents the pooled SMDs at 95% CI. **(A)** Forest plots; **(B)** Funnel plots. CysC, Cystatin C; SMD, standardized mean difference; CI, confidence interval.

The results of the meta-analysis revealed that the serum CysC levels of patients with hyperthyroidism were significantly higher than those of the controls (SMD: 1.79, 95% CI [1.34, 2.25]). The forest plots and funnel plots of serum CysC levels of patients with hyperthyroidism compared with those of controls are presented in **Figure 2**. The serum CysC levels of patients with hypothyroidism were significantly lower than those of the controls [SMD −0.59, 95% CI (−0.82, −0.36)]. The forest plots and funnel plots of serum CysC levels of patients with hypothyroidism compared with those of controls are presented in **Figure 3**.

### Sensitivity Analysis, Publication Bias, and Quality of Evidence

**Figure 4:**
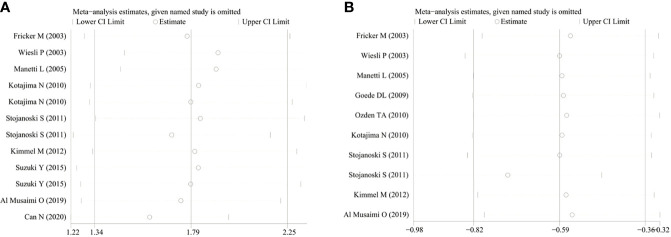
The sensitivity analysis results of serum CysC in patients with hyperthyroidism and hypothyroidism compared with controls. **(A)** Hyperthyroidism; **(B)** Hypothyroidism. CysC, Cystatin C.

A sensitivity analysis was performed to examine the influence of each study. We found no significant difference between the results of the sensitivity analysis and our previous estimates, indicating that our statistical results were relatively credible (**Figure 4**). Articles obtained from the databases were carefully and comprehensively searched. Egger’s test was also conducted to determine whether potential publication bias existed in the reviewed literature. While no publication bias was observed in the hyperthyroidism group (*P* > 0.05), some publication bias might have existed in the hypothyroidism group (*P* < 0.05). Using the approach recommended by the GRADE system, the certainty of the evidence for studies in the meta-analysis was evaluated as low or very low.

## Discussion

This meta-analysis evaluated the serum CysC levels of patients with thyroid diseases. Although some studies have investigated the association between serum CysC levels and thyroid diseases, their results were inconsistent. In this meta-analysis, 11 independent studies were included and analyzed. We concluded that the serum CysC levels of patients with hyperthyroidism and those with hypothyroidism were significantly higher and lower than those of the controls, respectively. The treatment of thyroid diseases also significantly affected the serum CysC levels, making CysC a potentially effective marker for monitoring the treatment of thyroid diseases.

CysC is a nonglycosylated protein with 120 amino acid residues. CysC production is more stable than creatinine production and is not affected by inflammation, bilirubin, and triglycerides, as well as sex, age, muscle mass, or diet. CysC, owing to its small molecular weight and positive charge, can freely pass through the glomerular filtration membrane and is almost completely reabsorbed in the proximal convoluted tubules. After reabsorption, it is completely catabolized and no longer returns to the blood circulation. Further, CysC is not secreted by the renal tubules and its production rate in the tissue is constant (1, 25). The kidney is the only organ that clears CysC in circulation. Blood CysC levels are determined by glomerular filtration, and inter-individual differences are small. The difference between the highest value in the population and the normal average value was <3–4 standard deviations. CysC is a new index that can reflect GFR with high specificity, good accuracy, and sensitivity compared with the creatinine clearance rate. It is an ideal endogenous marker that reflects the changes in GFR (26, 27).

Fricker et al. reported that an increase in the thyroid hormone content in the blood of patients with hyperthyroidism can promote the synthesis of Na^+^ - K^+^ - ATPase in many cells of the body, increase the basal metabolic rate and oxygen consumption, accelerate the cell renewal rate/metabolic rate, and promote the secretion of CysC by nucleated cells, thereby accelerating the production rate of CysC. Under the condition of normal renal function, the increase in serum CysC levels in patients with hyperthyroidism is not caused by renal injury but is related to increased basal metabolic rate and oxygen consumption (15).

Studies have shown that the serum transforming growth factor-β1 (TGF-β1) levels are significantly increased in patients with Graves’ disease and that there is a positive correlation between TGF-β1 and thyroid hormone levels. In addition, TGF-β1 levels decreased in patients with Graves’ disease after treatment. Studies have also reported that serum TGF-β1 levels in patients with hypothyroidism were significantly decreased. TGF-β1 stimulates vascular smooth muscle cells to secrete CysC. *In vivo*, TGF-β1 stimulated the secretion of CysC and increased the expression of CysC mRNA in HepG2 cells. Notably, T3 stimulated Hep-G2 cells to produce CysC in a dose-dependent manner (19). TGF-β1 treatment has been shown to upregulate CysC transcription in mouse embryonic cells and 3T3-L1 fibroblasts (28–30). Schmid et al. used a T3-responsive osteoblast cell line to investigate whether T3 stimulates the production of CysC *in vitro* and reported that T3 increased the expression and accumulation of CysC mRNA in the culture medium in a dose- and time-dependent manner. It was considered that the increased production of CysC induced by T3 may be related to an increased demand for cell metabolism and proteolysis control (31).

In our study, we noted that even mild thyroid dysfunction (subclinical hyperthyroidism/hypothyroidism) affected the serum CysC levels of patients. Due to the limited number of reports (only one paper) on subclinical thyroid diseases, we could not perform subgroup analysis. Thus, more studies on serum CysC levels and subclinical thyroid diseases are warranted.

### Strengths and Limitations

This meta-analysis firstly aimed to statistically evaluate serum CysC levels in patients with thyroid diseases. However, this study has some limitations. Due to the lack of case-control studies with a large sample population, most studies included in this meta-analysis were studies with a small sample population. Further, some studies did not use healthy controls controlled for body mass index. Different CysC detection methods were used among the studies, and the heterogeneity among studies on hyperthyroidism was high, partly due to different severities of hyperthyroidism being analyzed. These factors may have affected our results. Therefore, the results obtained herein should be interpreted cautiously, as further research is needed.

## Conclusion

To the best of our knowledge, this meta-analysis is the first to evaluate serum CysC levels in patients with thyroid diseases. Our findings suggest that thyroid function affects serum CysC levels and that serum CysC may be an effective marker to monitor the treatment of thyroid diseases. More high-quality studies are needed to better support the association between serum CysC levels and thyroid diseases.

## Data Availability Statement

The original contributions presented in the study are included in the article/**Supplementary Material**. Further inquiries can be directed to the corresponding authors.

## Author Contributions

XS designed the study. BS and XS searched databases and collected the data. HF and JX assessed the quality of the study. XS performed the analysis. HF and JX wrote the manuscript. All authors contributed to the article and approved the submitted version.

## Funding

This research was financially supported by the Natural Science Foundation of Jiangsu Province (grant No. SBK2020040002).

## Conflict of Interest

The authors declare that the research was conducted in the absence of any commercial or financial relationships that could be construed as a potential conflict of interest.

## Publisher’s Note

All claims expressed in this article are solely those of the authors and do not necessarily represent those of their affiliated organizations, or those of the publisher, the editors and the reviewers. Any product that may be evaluated in this article, or claim that may be made by its manufacturer, is not guaranteed or endorsed by the publisher.

## Supplementary Material

Supplementary Table 1 Preferred reporting items for systematic review and meta-analyses (PRISMA) checklist.

Supplementary Table 2 Full electronic search strategy for Pubmed.
