# Supplementary material for: Association Between Serum Cystatin C and Thyroid Diseases: A Systematic Review and Meta-Analysis
Source: Front Endocrinol (Lausanne). 2021 Nov 19;12:766516. doi: 10.3389/fendo.2021.766516 (PMC8639734; doi:10.3389/fendo.2021.766516)
Supplement: Supplementary Table 2 — Full electronic search strategy for Pubmed. [file Table_2.docx]

The full electronic search strategy for Pubmed: ("cystatin c"[Title/Abstract] OR "CysC"[Title/Abstract]) AND ("thyroid disease"[Title/Abstract] OR "thyroid function"[Title/Abstract] OR "hypothyroidism"[Title/Abstract] OR "hyperthyroidism"[Title/Abstract]). I also upload the search strategy in the supplementary file.
